# Structural Features of Connective Tissue Formed around Resin Implants Subcutaneously Embedded in Dairy Cows

**DOI:** 10.3390/ani13233700

**Published:** 2023-11-29

**Authors:** Yuka Katayama, Osamu Ichii, Teppei Nakamura, Keita Yanase, Masaya Hiraishi, Takashi Namba, Yuki Otani, Teppei Ikeda, Erika Tsuji, Natsuko Tsuzuki, Ken Kobayashi, Yasuhiro Kon, Takanori Nishimura

**Affiliations:** 1 Laboratory of Agrobiomedical Science, Faculty of Agriculture, Hokkaido University, Sapporo 060-8589, Japan; ry05140514@gmail.com (Y.K.); ichi-o@vetmed.hokudai.ac.jp (O.I.); nakamurate@vetmed.hokudai.ac.jp (T.N.); keita.yanase@furukawaelectric.com (K.Y.); m-hiraishi@vetmed.hokudai.ac.jp (M.H.); 2 Laboratory of Cell and Tissue Biology, Research Faculty of Agriculture, Hokkaido University, Sapporo 060-8589, Japan; kkobaya@agr.hokudai.ac.jp; 3 Laboratory of Anatomy, Department of Basic Veterinary Sciences, Faculty of Veterinary Medicine, Hokkaido University, Sapporo 060-0818, Japan; namba.t28@gmail.com (T.N.); yukiotani35@vetmed.hokudai.ac.jp (Y.O.); y-kon@vetmed.hokudai.ac.jp (Y.K.); 4 Laboratory of Animal Science and Medicine, Department of Applied Veterinary Sciences, Faculty of Veterinary Medicine, Hokkaido University, Sapporo 060-0818, Japan; 5 Furukawa Electric Co., Ltd., Tokyo 100-8322, Japan; 6 BRAST Livestock Clinic, BRAST Inc., Sapporo 004-0033, Japan; t.ikeda@brast.hokkaido.jp; 7 Department of Biological Safety Research, Chitose Laboratory, Japan Food Research Laboratories, Chitose 066-0052, Japan; tsujie@jfrl.or.jp (E.T.); tsuzukin@jfrl.or.jp (N.T.)

**Keywords:** cow, connective tissue, foreign body reaction, inflammation, fibrosis

## Abstract

**Simple Summary:**

The relation between implant components, foreign body reactions to implant materials, and the formation of connective tissue (CNT) in host animals is crucial for developing effective xenotransplantation techniques. This study demonstrated that dairy cows produce several large, dense, collagen-based CNTs following subcutaneous implantation of acrylic resin-based implants. We elucidated the process of CNT formation within the implants. The implants used in this study, comprising an outer cylinder, a core rod, and caps at both ends, were composed of acrylic resin created using a three-dimensional (3D) printer. No abnormalities, such as chronic inflammation, were observed. An advantage of 3D printing technology in the medical field is its ability to produce implants with complex shapes for surgical applications. Therefore, the combination of acrylic resin and 3D printer design holds promise for researching foreign body reaction and clinical applications in future studies.

**Abstract:**

Foreign body reactions (FBRs) are inadvertently observed in invading or artificially embedded materials, triggering inflammation and subsequent fibrotic processes to occur in situ. Here, we assessed the spatiotemporal formation of connective tissue around implanted materials to establish a technique using connective tissue formed by FBRs as xenografts. An acrylic resin implant, comprising a columnar inner rod and a tubular outer cylinder (OC) with several slits, was embedded in adult dairy cows. Tissues formed in the inner rod and OC groups were histologically analyzed at weeks 2, 4, 8, and 12. Edematous tissues with non-collagenous fibers formed for 2 weeks and showed increased cellularity after 4 weeks. The weight, thickness, amounts of total protein, collagen, DNA, and quantitative scores of α-smooth muscle actin-positive myofibroblasts or elastic fibers notably increased after 8 weeks, with condensed collagen fibers showing orientation. Inflammatory cells were primarily localized in tissues close to the OC, and their numbers increased, with the count of CD204+ cells peaking at 8 weeks and declining at 12 weeks. The count of Ki67+ proliferating cells slightly increased in tissues close to the OC; however, the number and lumen of CD31+ vessels increased. These results may help understand FBR-related tissue remodeling.

## 1. Introduction

Wounded tissue healing involves four phases: hemostasis, inflammation, proliferation, and remodeling, with macrophages (Mφ), specifically inflammatory M1Mφ, anti-inflammatory M2Mφ, and hybrid M1/M2 Mφ, playing crucial roles [1,2]. Inflammation leads to the accumulation of M1Mφ and neutrophils, whereas M2Mφ and activated fibroblasts (myofibroblasts) accumulate during the proliferative phase as inflammation subsides. Myofibroblasts can produce collagen, which makes up the extracellular matrix (ECM), which is essential for fibrogenesis and tissue remodeling [3,4]. A higher M2Mφ/M1Mφ ratio during healing is associated with ECM production [5,6].

Mφ plays key roles in foreign body reactions (FBRs) occurring in invading or embedded materials [7]. FBRs involve cell infiltrations of various immune cells, such as Mφ, T cells, B cells, and granulocytes [8,9]. Mφ primarily mediates FBRs via their phagocytic function, producing factors such as transforming growth factor beta (TGF-β), which is crucial for myofibroblast differentiations leading to fibrosis around the foreign body [8,10].

The widespread utilization of medical devices has resulted in adverse FBR-related biological events [11]. The specific characteristics of the implant material play a crucial role in influencing FBRs, impacting the behavior of immune cells and the thickness of fibrous capsules formed around the implant. Intravenously implanted glucose biosensors may exhibit reduced sensitivity due to FBRs, leading to inaccurate reflection of glucose levels [12]. Therefore, biomaterials must possess high biocompatibility to minimize the impact of FBRs [13]. Notably, polymethyl methacrylate acrylic resins find common use in orthopedics and dentistry owing to their recognized biocompatibility, reliability, ease of manipulation, and low toxicity [14].

The use of biomaterials has led to an innovative technique that utilizes connective tissues (CNTs) for graft creation induced by FBRs via artificial implantation of materials into subcutaneous regions over several months [15]. This technique has been successful in previous studies, such as the use of bovine-derived CNTs for canine abdominal wall and pulmonary artery patches, resulting in autologous tissue regeneration within a few months [16]. FBRs trigger histopathological changes around the embedded materials, offering a unique and beneficial approach to transplant procedures. In our previous study in mice, we demonstrated inflammation and CNT development until day 21, followed by inflammation stabilization and CNT convergence until day 70 in the tissues surrounding the artificially embedded silicone [17]. However, the time courses of other species and biomaterials remain unclear.

This study focused on fibrosis-based FBRs in acrylic resin implants used in dairy cows. While previous clinical studies have investigated acrylic resin FBRs, no study has examined the FBR process, specifically around implants. The goal of this study is to establish a unique method for producing substantial and large CNT grafts, aiming to enhance the economic value of retired cows in the future. Therefore, this study clarified the spatiotemporal changes in CNT formed around implants created using cost-effective three-dimensional (3D) printing and embedded in dairy cows for 12 weeks. Accumulating studies exploring the relation among implant components, FBRs, and CNT formation in host animals are pivotal for future advancements in xenotransplantation techniques.

## 2. Materials and Methods

### 2.1. Animals

Four female Holstein cows (34, 37, 46, and 52 months old; approximately 600 kg body weight) were housed in a loose barn and grazed at the Facility for Farm Animals, Faculty of Veterinary Medicine, Hokkaido University (Sapporo, Japan). These cows were in a dry period and maintained in a microbiologically conventional environment with a 12 h light–dark cycle. All animal experiments were conducted following approval from the Institutional Animal Care and Use Committee of the Faculty of Veterinary Medicine, Hokkaido University (approval No. 19-0104, dated 26 September 2019). The handling of animals adhered to the guidelines outlined in the Guide for the Care and Use of Laboratory Animals, Faculty of Veterinary Medicine, Hokkaido University (approved by the Association for Assessment and Accreditation of Laboratory Animal Care International).

### 2.2. Preparation of Implants

**Figure 1:**
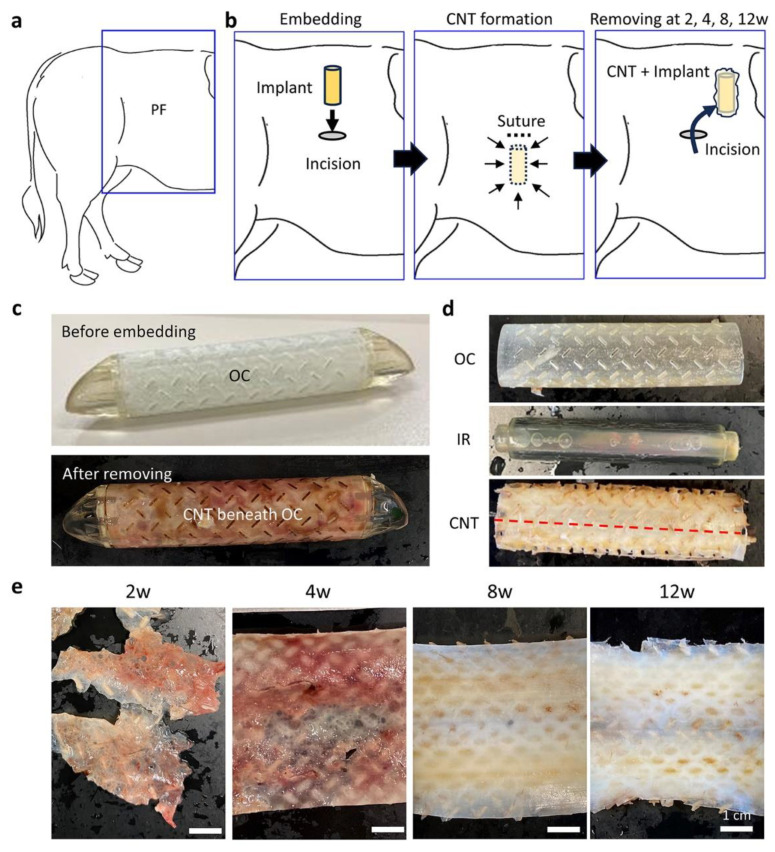
Summarized experimental procedure and connective tissues formed around the subcutaneously embedded resin implant. (**a**) Paralumbar fossa (PF) of a cow for experimental surgery. (**b**) Embedding of implant in the incised area in PF; connective tissue (CNT) formation (arrows) after closure by suture; and removal of the implant with CNT at 2, 4, 8, and 12 weeks (w) after surgery. (**c**) Resin implant used in this study before and after embedding. Brown color indicates the CNT beneath the outer cylinder (OC). (**d**) Removed implant separated into the OC, inner rod (IR), and CNT formed between OC and IR. For analysis, the CNT was cut and opened according to the red dotted line. (**e**) Appearance of the sheet-like CNT removed for different embedding periods.

The implants were prepared by assembling three acrylic resin parts (Furukawa Electric. Co., Ltd., Tokyo, Japan). Blueprints for the implants were created using computer-aided design software (Solidworks 2020, Waltham, MA, USA) and fabricated using a 3D printer (Objet 30 Prime, Stratasys Ltd., Eden Prairie, MN, USA). These processes were performed by Furukawa Electric. Co., Ltd. The columnar inner rod (outer diameter: 15 mm; length: 80 mm) had a smooth surface. The tubular outer cylinder (outer diameter: 20 mm, inner diameter: 17 mm, length: 80 mm) had several slits (144, 1 mm × 4 mm). The inner and outer cylinders were connected using caps (Figure 1), and the gap between the inner and outer cylinders was 1 mm. This space is designed to be filled with CNTs. The weight of the assembled implant was 44 g. Implants were autoclaved at 121 °C for 15 min immediately before implantation surgery.

### 2.3. Surgical Embedding of the Resin Implants

Lumbar epidural anesthesia, which targeted the inner arcuate spaces between the 13th thoracic, 1st lumbar, or 2nd lumbar vertebrae, was administered to the cows. This location was altered based on the ease of puncture and reaction of the cows. A mixture of 4.5–5.5 mL of lidocaine (0.150–0.183 mg/kg BW; Nagase Medicals Co., Ltd., Hyogo, Japan) and 0.5 mL of xylazine (0.0167 mg/kg BW; Elanco Japan K. K., Tokyo, Japan) was injected into the epidural space using a needle (16 G × 120 mm, Hakko Co., Ltd., Nagano, Japan; 18 G × 130 mm, Fujihira Industry Co., Ltd., Tokyo, Japan). Subcutaneous infiltration anesthesia (lidocaine, 20 mg/mL) was administered as necessary. The surgical fields at the paralumbar fossa or skin area on the fascia of the external oblique abdominal muscle were washed with a brush and a liquid soap and disinfected with a combination of 7.5% isodine scrub solution (Mundipharma K. K., Tokyo, Japan), 10% polyvinylpyrrolidone isodine solution (Fujita Pharmaceutical Co., Ltd., Tokyo, Japan), and 70% ethanol, or a single spray of Klorus disinfectant water (8000 ppm, PURGATIO Inc., Tokyo, Japan). After skin scission, the prepared resin implants were embedded between the cutaneous muscle of the trunk and the external oblique abdominal muscle, with a maximum of six implants per side (Figure 1a,b). The incised wounds were closed using absorbable sutures (Opepolix, #HC5022GV90-KN2, Alfresa Pharma K. K., Osaka, Japan). Flunixin meglumine (2 mg/kg BW; MSD Animal Health K.K., Tokyo, Japan) and ceftiofur (6.6 mg/kg BW; Zoetis Japan K.K., Tokyo, Japan) were administered intravenously and subcutaneously as an analgesic and antibiotic, respectively.

### 2.4. Surgical Removal of the Resin Implant and Sample Preparations

The implants were removed from the cows 2, 4, 8, and 12 weeks after embedding. The numbers and positions of the embedded implants are summarized in Supplementary Figure S1. The cows were briefly anesthetized, operated on, and subjected to postoperative management using the same method as that used for the embedding operation. The CNTs that formed between the inner and outer cylinders were carefully separated from the implants, opened along the long axis (Figure 1c–e), and weighed. We obtained one sheet-like tissue from one implant, which was then divided and fixed in 10% neutral buffered formalin (NBF), 2.5% glutaraldehyde/0.1 M phosphate buffer, or saline solution for histological, ultrastructural, or physical analysis, respectively.

### 2.5. Biochemical Analysis

The amounts of total protein, collagen, and DNA were measured in the NBF-fixed tissues. Three sections of size 0.5 × 1.0 cm were cut from the tissues, wiped dry, and weighed. Six molar hydrochloric acid was added to the weighed tissues and heated at 95 °C for 20 h. The supernatant was collected via centrifugation. Total protein and collagen were quantified using colorimetric assays (Total Protein Assay Kit and Total Collagen Assay Kit; QuickZyme Biosciences, Leiden, The Netherlands). DNA was extracted from NBF-fixed tissues as previously described, with minor modifications [18,19]. Briefly, 1.0 mL of Tail Lysate (Nacalai Tesque, Kyoto, Japan) was added to the weighed tissue and heated at 80 °C for 4 h. The tissues were digested with proteinase K at 56 °C overnight, and then treated at 90 °C for 30 min to inactivate proteinase K. All DNA samples were extracted using the phenol/chloroform/isoamyl alcohol method, purified by ethanol precipitation, and dissolved in nuclease-free water. DNA concentrations were measured using a NanoDrop One (Thermo Fisher Scientific, Waltham, MA, USA).

### 2.6. Histological Analysis

Four to eight pieces of NBF-fixed tissue were embedded in paraffin and cut into sections (4 μm thick), including the cross-sectional surface of the CNTs. The deparaffinized sections were stained with hematoxylin and eosin (H&E) or Elastica Van Gieson (EV).

Immunohistochemistry (IHC) for the representative cell markers, including α-smooth muscle actin (α-SMA), CD20, CD3, nitric oxide synthase 2 (NOS2), Mφ scavenger receptor 1 (also known as CD204), allograft inflammatory factor 1 (also known as IBA1), marker of proliferation Ki-67 (also known as KI67), and platelet and endothelial cell adhesion molecule 1 (also known as CD31), was performed to detect smooth muscle cells, pan B cells, pan T cells, M1Mφ, M2Mφ, pan Mφ, proliferating cells, and vascular endothelial cells, respectively. IHC was performed according to a previously described method using deparaffinized sections [17]. Finally, the sections were stained with hematoxylin. Details of the antibodies used, antigen retrieval conditions, and blocking procedures are presented in Table 1.

### 2.7. Histoplanimetry

All sections were converted into virtual slides using a Nano Zoomer 2.0 RS (Hamamatsu Photonics Co., Ltd., Shizuoka, Japan).

#### 2.7.1. Thickness of the CNTs

Using the data from the H&E-stained sections, a line perpendicular to the cross-section of the tissue was drawn, and the length was measured along this line from the surface in contact with the outer sleeve to the surface in contact with the core rod. This measurement was performed using NDP.view2 (Hamamatsu Photonics Co., Ltd.) on 29 samples with 24–40 random locations on each sample.

#### 2.7.2. Elastic Fiber Area and Positive Cell Area in the IHC

For the assessment of elastic fiber area in EV staining or the positive cell area in IHC, the virtual slides were converted into binary images using Photoshop CC 2018 (Adobe, San Jose, CA, USA). In this process, the stained areas were converted to white, while other colored areas were converted to black. The total area or the white area was then calculated using ImageJ (NIH, Bethesda, MD, USA). Subsequently, the ratio of the white area to the total area (%) was expressed as the quantified values for elastic fiber areas or IHC-positive areas, respectively. The quantification was carried out across 29 samples, with 4 locations selected randomly for each sample in the case of elastic fibers and 9–32 locations for IHC.

### 2.8. Scanning Electron Microscopy

The harvested tissue was sectioned transversely (2–3 mm thick) and fixed in 2.5% glutaraldehyde for 4 days. After washing with distilled water, the tissue was immersed in 1% tannic acid solution for 12 h and then immersed and fixed in 1% osmium tetroxide solution for 1 h. After dehydration in an ethanol series, samples were lyophilized using tert-butyl alcohol. The dried specimens were fixed to a specimen base with carbon paste, deposited with platinum–palladium and observed under a scanning electron microscope (JSM-6301F; JEOL, Tokyo, Japan) at an acceleration voltage of 8–10 kV.

### 2.9. Statistical Analysis

Data are expressed as mean ± standard deviation and were analyzed statistically by nonparametric methods using IBM SPSS Statistics 28.0.1.0 (142) (IBM; Armonk, NY, USA). Data were analyzed using nonparametric statistical methods. The Smirnov–Grubbs test was used to test for and exclude outliers from the data obtained for each measurement, followed by the Kruskal–Wallis test. Multiple comparisons were performed using Scheffé’s method when significant differences were observed (*p* < 0.05). 

## 3. Results

### 3.1. CNTs Formed around the Embedded Resin Implant

At 12 weeks after embedding the resin implant, firm milky white and tube-shaped CNTs formed between the inner and outer cylinders (Figure 1c–e). The size of the CNTs after excision was approximately 60 × 80 mm, and several brown spots corresponding to the resin implant slits were observed upon gross examination (Figure 1e). In contrast, the tissues that formed between 2 and 4 weeks were reddish brown, fragile, and thin, while those formed between 8 and 12 weeks tended to be solid.

### 3.2. Histology of CNTs Formed around the Embedded Resin Implant

**Figure 2:**
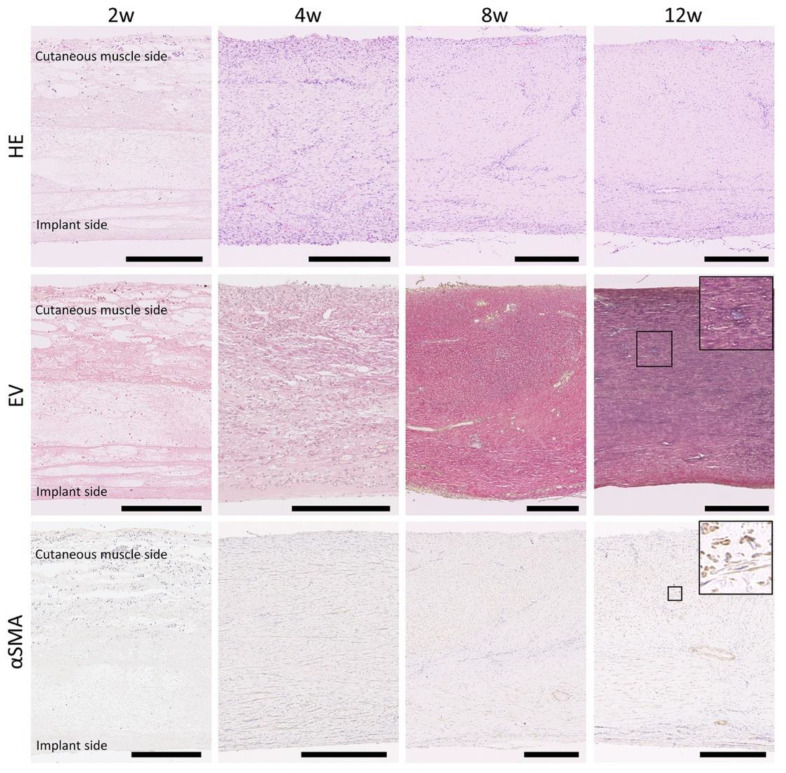
Histology of connective tissues formed around resin implant. Cross-sections of the connective tissues. H&E: hematoxylin and eosin staining; EV: Elastica Van Gieson staining; SMA: Immunohistochemistry for smooth muscle actin; w: weeks after embedding. Bars = 250 μm.

In the histological sections stained with H&E at 2 weeks (Figure 2), loose and fragile tissues were observed with scarce cells, and several edematous and cell-free areas occupied the tissues. At 4 weeks, the tissue thickness was almost the same as that at 2 weeks, but the number of cells and density of the CNTs tended to increase. As shown in Supplementary Figure S2, fibrin fibers were detected in the edematous area at 2 and 4 weeks using phosphotungstic acid–hematoxylin staining. The thickness, cell number, and density of the CNTs increased remarkably after 8 and 12 weeks, and cellular localization differed among the tissue positions, with the superficial and middle layers indicating tissues close to the implant and other tissues, respectively; the superficial layers tended to have more cells than the middle layer. Well-developed fiber structures were observed after 12 weeks. EV staining revealed the localization of elastic fibers in the CNTs; they were scarcely observed at 2 and 4 weeks and their proportion slightly increased from 8 to 12 weeks. In IHC, αSMA^+^ cells were detected in the fiber structures at 8 and 12 weeks and were observed across the organization.

### 3.3. Fiber Arrangement in the CNTs Formed around the Embedded Resin Implant

**Figure 3:**
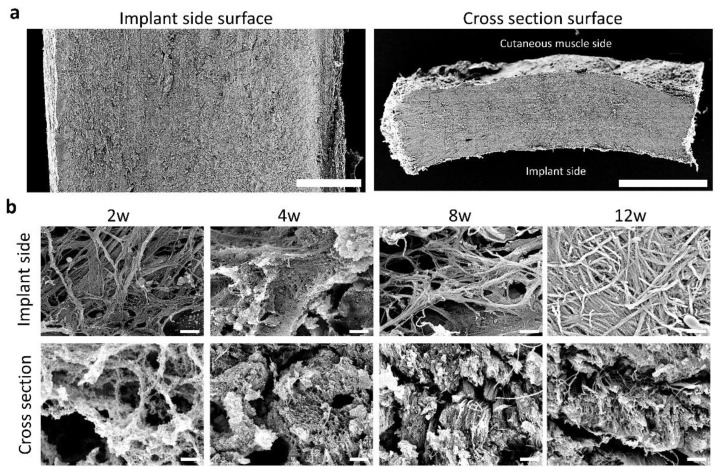
Collagen fiber arrangements in the connective tissues formed around resin implant. Scanning electron microscopy images. (**a**,**b**) Surfaces of the implant side and cross-sections of the connective tissues at a low and high magnifications, respectively. Bars = 1 mm (**a**), 2 μm (**b**). w: weeks after embedding.

As shown in Figure 3a, the fiber arrangement was observed at the surface of the implant side or cross-section using scanning electron microscopy. On the implant-side surface (Figure 3b), the fibers were reticularly arranged, and the space between the fibers was wide between 2 and 8 weeks. At 12 weeks, a dense reticular arrangement of fibers was observed. On the cross-sectional surface, loose fiber bundles were observed at 2–4 weeks, which became dense at 8 and 12 weeks.

### 3.4. Infiltration of Immune Cells to the CNTs Formed around the Embedded Resin Implant

**Figure 4:**
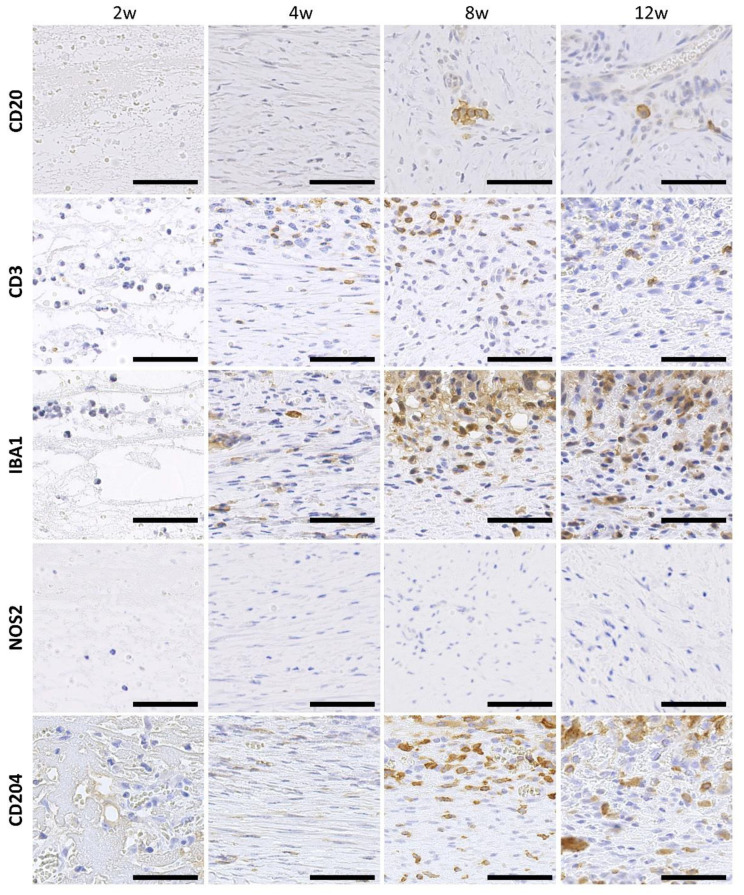
Infiltration of immune cells to the connective tissues formed around the embedded resin implant. Cross-sections of the connective tissues. Immunohistochemistry for CD20, CD3, IBA1, NOS2, and CD204. w: weeks after embedding. The upper direction of panels shows the superficial layers. Bars = 50 μm.

As shown in Figure 4, very few CD20+ B cells were observed during the observation period. A few positive cells were observed in the middle layers of the tissue, most of which formed clusters. CD3+ T cells were relatively few in number. On the other hand, IBA1+ Mφ were observed in all samples and were abundant from 4 weeks onward. Only a few NOS2+ M1Mφ were found in all samples (Supplementary Figure S3). CD204+ M2Mφ were abundant at 8 and 12 weeks. These immune cells tended to be localized in the superficial layers of the tissue.

### 3.5. Proliferation Activity and Angiogenesis in the CNTs Formed around the Embedded Resin Implant

**Figure 5:**
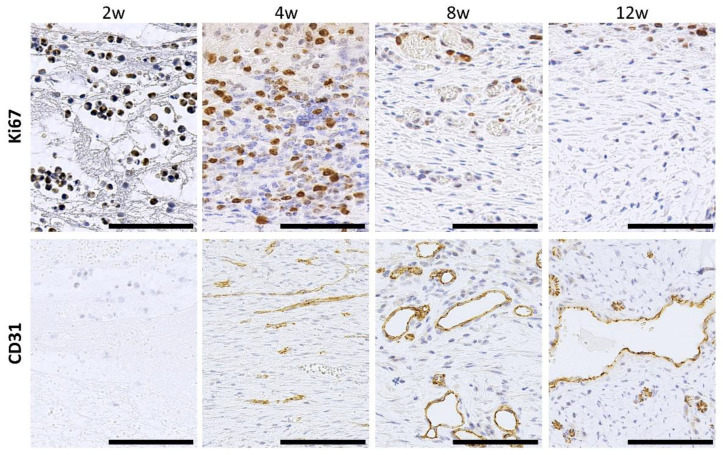
Proliferation activity and angiogenesis in the connective tissues formed around the embedded resin implant. Cross-section of connective tissues. Immunohistochemistry of Ki67 and CD31. w: weeks after embedding. Bars = 100 μm.

Ki67+ proliferating cells were present throughout the observation period and unevenly localized in the superficial layers (Figure 5). CD31+ endothelial cells were rarely observed at 2 weeks, but positive reactions were observed from 4 weeks onward. At 4 weeks, samples showed linear positive reactions without lumen formation; however, vascular lumen formation was clearly observed at 8 weeks, and expansion of the lumen was observed at 12 weeks.

### 3.6. Summary of Quantitative Changes in the CNT

Table 2 summarizes the results of the quantitative analyses. As for the indices of cellularity and amount of collagen in CNTs, the weight and amount of total protein, collagen, and DNA in CNTs increased with time. The values at 8 and 12 weeks were significantly higher than those at 2 and 4 weeks in terms of the weight and amounts of total protein and collagen. Significant differences in the amount of DNA were observed between 8 and 2 weeks and between 12 and 2 weeks or 4 weeks. 

For those results regarding connective fiber development, tissue thickness and αSMA^+^ area at 8 and 12 weeks were significantly higher than those at 2 and 4 weeks. The elastic fiber area also increased with time and was significantly higher at 8 and 12 weeks than at previous time points. 

For cell infiltration, the CD20+ cell area was slightly increased at 8 and 12 weeks and significantly increased at 4 and 8 weeks. The IBA1+ cell area showed an increasing trend during the observation period, with no significant differences between the time points. The NOS2+ cell area did not change during the observation period and was low throughout. The CD204+ cell area increased from 4 to 8 weeks, peaked at 8 weeks with significant differences compared to that at other time points, and decreased at 12 weeks. The CD31+ cell area for angiogenesis tended to increase from 2 to 12 weeks, and the value at 12 weeks was significantly higher than that at other time points. The CD3+ cell area or Ki67+ cell area did not exhibit significant changes during the observation period.

## 4. Discussion

In this study, we demonstrated that dairy cows produce several dense, collagen-based, large CNTs following the subcutaneous implantation of acrylic resin-based implants. We also clarified the process of CNT formation in the implants. The implants used in this study were composed of acrylic resin created using a 3D printer. Acrylic resin is used to create dentures and for cranioplasty because of its high biocompatibility [20,21]. In the present study, no abnormalities, such as chronic inflammation, were observed. An advantage of 3D printing technology in the medical field is its ability to produce implants with complex shapes for use in surgical fields, such as orthopedics and cranioplasty [22]. An implant consisting of an outer cylinder, a core rod, and caps at both ends was easily fabricated using a 3D printer after the design. Therefore, the combination of acrylic resin and 3D printer design may be useful for FBR research and clinical applications in future studies.

This study aimed to develop a CNT-based graft while considering animal ethics. Dairy cows destined for slaughter were used to create artificial CNTs. We also considered the future applications and ethical aspects of animal use. There is potential to find additional economic value in retired cattle. Moreover, we anticipated that the larger the animal in which the implant was placed, the stronger the CNTs, the larger the size of each tissue, and the higher the number of tissue bodies that could be produced simultaneously. The obtained CNT sheets were notably larger than those obtained from smaller animals, such as rodents, rabbits, or dogs. In a previous study using goats as experimental animals, each individual produced three to seven sheets of tissue (5 × 7 cm) [23]. However, in the current study, six pieces of tissue measuring approximately 62 × 80 mm were produced from a single bovine body. Additionally, the Young’s modulus of CNTs produced in rabbits is less than half of that in bovines, and herniation occurs in 21% of patch tests on the diaphragm [24], indicating the strength of the bovine CNT sheets. Nevertheless, the substantial weight of cows sometimes distorts certain implants, which may affect the thickness of CNTs. This may necessitate the consideration of more robust implants in large animals.

In the present study, the weight, total protein content, collagen content, and percentage of elastic fibers per unit area of CNTs increased notably from the fourth to the eighth week. Furthermore, the tissues obtained in this study were the thickest at 8 weeks, approximately 0.7 mm for a 1 mm gap between the outer cylinder and the core rod. A previous study in cows reported that the thickness of the tissue formed 8–12 weeks after implantation was approximately 80% of the implant gap, and the results of this study are similar to those of a previous study [25]. Several studies used rabbits, dogs, goats, and cows, in which the CNT preparation period was 2–3 months. Collagen-based CNTs were formed inside the implant and functioned in the recipient’s body after transplantation [25,26,27,28]. From these results, we concluded that stable CNTs could be created approximately 2–3 months after implantation, regardless of the animal species.

M1Mφ evoke inflammatory responses, and M2Mφ are involved in anti-inflammatory responses or fibrosis [1,2,3,4,5,6]. In the present study, M1Mφ were scarce until 12 weeks, whereas the number of M2Mφ increased notably from 4 to 8 weeks. This result suggests two possibilities: one is that the initial inflammatory response by M1Mφ had already converged by 2 weeks; the other is that the high biocompatibility of the implants induced minimal inflammation inside the implants. With a significant increase in M2Mφ number, the α-smooth muscle actin cell area, tissue thickness, weight, collagen content, and elastic fiber area also increased notably from 4 to 8 weeks. These changes could be due to enhanced CNT formation by M2Mφ.

Histological differences were observed between the superficial and middle layers. The superficial layers contained more cells than the middle layers. Regarding similar morphological maldistribution, a study in which CNTs were created by implanting an implant under canine skin for 4 weeks found that type III collagen, whose level increases early in tissue repair, is present in the tissue facing the outer edge of the implant [29]. In the present study, IBA1+ M1/M2φ, CD204+ M2φ, and Ki67+ proliferating cells were observed in the superficial layer facing the implant but not in the middle layer. These results suggested that the superficial layer created with the porous implant proceeded into the inflammatory and proliferative phases, in contrast to the middle layer.

During the early phase of the wound healing process following tissue damage caused by implant insertion, the interaction between blood and the implant surface results in the adsorption of plasma proteins, forming a provisional matrix that serves as an adhesive for inflammatory cells [30]. Fibrin (derived from fibrinogen) promotes macrophage adhesion [31]. In a similar CNT produced subcutaneously in dogs, sparse tissue composed of fibrin was formed 2 weeks after implantation [29]. Fibrin fibers were present in 2- and 4-week tissues in the present study (Supplementary Figure S2). Fibrin polymers promote macrophage fusion to form foreign body giant cells [32]. Mononuclear Mφ and foreign body giant cells secrete factors that promote CNT formation in myofibroblasts [33]. In particular, TGF-β is important for the conversion of fibroblasts to myofibroblasts, a critical process for CNT formation around the implant [8].

Elastic fibers are involved in wound healing via TGF-β signaling and integrin-mediated cell adhesion [34]. Major elastic fiber proteins include tropoelastin, fibrillin-1, and fibrin-5 [35]. Experiments in mice have shown that elastic fibers are not necessary in the early stages of wound healing but play a supportive role in the remodeling of dermal structures [36]. In the present study, a marked increase in the elastic fiber area was also seen after 4 weeks following treatment with collagen fibers, indicating a crucial role of elastic fibers in FBR-related CNTs.

Angiogenesis, a wound healing event, is important for tissue remodeling [37]. Mouse experiments have shown that the endothelial calpain system involved in wound healing affects not only neovascular sprouting but also fibroblast activation by endothelial cells and subsequent fiber formation [38]. In this study, the number of CD31+ endothelial cells showed an increase over time consistent with the increase in SMA+ cell number, collagen content, and elastic fiber area, indicating an association between angiogenesis and CNT formation in FBR. In addition, neovascularization was rarely observed in the superficial layer but was observed more frequently in the middle layer. This result suggests that angiogenesis was inhibited in the superficial layer due to anti-angiogenic factors; in particular, thrombospondin 2 is known to be highly expressed at the implant interface and is associated with fibroblasts in the vicinity of the capsule [39,40].

In summary, CNTs were formed without any abnormalities, such as chronic inflammation, by the subcutaneous implantation of bovine skin in an implant created using acrylic resin and 3D printing. The use of bovines enables the creation of many tissue bodies in a single implantation surgery, and the tissue bodies after 8 weeks may be used as CNT-rich tissue in transplantation. In a previous study, in which bovine-derived CNTs preserved in 70% ethanol were implanted into the abdominal wall of a dog without decellularization, inflammation was observed 1 month after implantation; however, 3 months later, the CNTs were replaced with autologous collagen-bonded tissue without rejection [16]. Although the remaining cells in the graft could induce an immune response in the recipient, the number of cells in the tissue remained high after 12 weeks. Therefore, for the clinical application of FBR-related CNTs as grafts, the effects of the bioreactor, recipient animal species, transplantation site, transplanted tissue size, and decellularization treatment should be examined in future studies.

## 5. Conclusions

**Figure 6:**
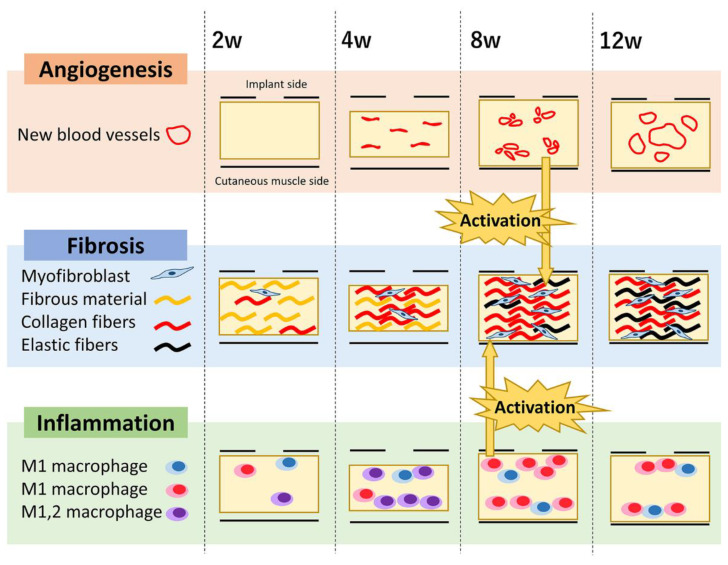
Connective tissue formation process. Fiber-like material was produced first, and collagen fibers were produced from the slit area for 4 weeks. At 8 weeks, neovascular vessels formed a lumen, fibroblasts were activated by vascular endothelial cells and Mφ, and collagen content and elastic fiber area markedly increased.

In this study, we assessed CNT formation around implanted materials to establish a technique for using CNT formed by FBRs as xenografts. Our results showed unique implant-related CNT formation. The CNT formation process is illustrated in Figure 6. Following implantation, an edematous-like structure containing non-collagenous fibers partially composed of fibrin was formed in its internal space for 2 weeks. Further, angiogenesis was virtually absent, and cellular infiltration was minimal. From 2 to 4 weeks, the number of myofibroblasts and macrophages in the CNTs simultaneously increased, forming a vascular-like structure with no lumen. The number of collagen fibers increased significantly with the elastic fiber area from 4 weeks. At 8 weeks, CNT formation stabilized and neovascular vessels with visible lumens were observed. The tissues at 12 weeks showed inflammatory cells in the superficial layers; however, the neovascular lumen was enlarged in the middle section. These results may contribute to our understanding of FBR-related tissue remodeling. 

Further studies on the relation between the components of implants, FBRs to the implanted materials, and CNT formation in host animals are crucial for developing xenotransplantation techniques in the future.

## Figures and Tables

**Table 1:** Summary of immunohistochemistry conditions. α-SMA: α-smooth muscle actin; NOS2: nitric oxide synthase 2.

Antibody	Ki67	α-SMA	CD20	IBA1	CD3	NOS2	CD31	CD204
Host	Rabbit							Mouse
Dilution	1:1500	1:100	1:500	1:3000	1:100	1:500	1:100	1:300
Source	Abcam, Cambridge, UK			FUJIFILM Wako, Osaka, Japan	Nichirei, Tokyo, Japan	Santacruz, CA, USA	Abcam, Cambridge, UK	TransGenic, Fukuoka, Japan
Antigen retrieval	CB, 105 °C, 20 min						VT, 105 °C, 20 min	CB, 105 °C, 20 min
Blocking serum	10% normal goat serum (cat. #: 426042, Nichirei)							10% normal rabbit serum (cat. #: 426052, Nichirei)

VT: HistoVT One (Nacalai Tesque, Kyoto, Japan), CB: 0.01 M citrate buffer (pH 6.0).

**Table 2:** Summary of quantitative changes in the CNT.

Indices		Parameters	Weeks after Embedding			
			2	4	8	12
Cellularity and collagen amount		Weight (mg/cm^2^)	31.01 ± 8.06 ^a^	49.08 ± 18.52 ^a^	79.85 ± 14.22 ^b^	83.48 ± 23.41 ^b^
		Total protein (mg/cm^2^)	4.30 ± 0.53 ^a^	6.53 ± 4.55 ^a^	13.55 ± 3.57 ^b^	15.75 ± 5.33 ^b^
		Collagen (mg/cm^2^)	0.02 ± 0.01 ^a^	0.64 ± 1.18 ^a^	3.83 ± 1.91 ^b^	5.28 ± 2.38 ^b^
		DNA (μg/cm^2^)	36.01 ± 21.51 ^a^	77.51 ± 105.46 ^ab^	150.62 ± 54.07 ^bc^	186.20 ± 65.54 ^cd^
Connective fiber development		Tissue thickness (μm)	448.39 ± 140.66 ^a^	348.38 ± 168.56 ^a^	708.13 ± 180.64 ^b^	615.84 ± 157.96 ^b^
		Elastic fiber area (%)	0.10 ± 0.09 ^a^	0.47 ± 0.63 ^a^	2.22 ± 1.03 ^b^	4.48 ± 0.63 ^c^
		αSMA+ cell area (%)	0.05 ± 0.02 ^a^	0.20 ± 0.21 ^a^	2.93 ± 0.88 ^b^	2.54 ± 1.83 ^b^
Lymphocytes	B cells	CD20+ cell area (%)	0.03 ± 0.01 ^a^	0.05 ± 0.04 ^a^	0.15 ± 0.08 ^b^	0.14 ± 0.11 ^b^
	pan T cells	CD3+ cell area (%)	0.16 ± 0.05	0.17 ± 0.06	0.54 ± 0.34	0.77 ± 0.80
Mφ	M1/M2-type	IBA1+ cell area (%)	0.54 ± 0.27	1.54 ± 1.75	1.41 ± 0.78	1.86 ± 1.36
	M1-type	NOS2+ cell area (%)	0.03 ± 0.01	0.23 ± 0.20	0.12 ± 0.11	0.21 ± 0.14
	M2-type	CD204+ cell area (%)	0.18 ± 0.11 ^a^	0.22 ± 0.09 ^a^	1.73 ± 0.86 ^b^	1.13 ± 1.15 ^a^
Proliferation		Ki67+ cell area (%)	0.51 ± 0.31	0.97 ± 0.97	0.82 ± 0.64	0.73 ± 0.26
Angiogenesis		CD31+ cell area (%)	0.03 ± 0.01 ^a^	0.95 ± 1.38 ^a^	1.70 ± 1.29 ^a^	2.43 ± 0.94 ^b^

Value = mean ± SD, standard deviation. Kruskal–Wallis test was followed by Scheffe’s method (*p* < 0.05). Significant differences were observed between the different letters. n = 8 (2 weeks), 9 (4 weeks), 6 (8 and 12 weeks).

## Data Availability

Data supporting the findings of this study are available from the corresponding author upon request.

## Supplementary Materials

The following supporting information can be downloaded from https://www.mdpi.com/article/10.3390/ani13233700/s1, Figure S1. Cows and places of embedded implants used in this study Figure S2: Fibrin fibers of connective tissue formed around resin implants Figure S3: Immunohistochemistry of NOS2.
