# Supplementary material for: Structural Features of Connective Tissue Formed around Resin Implants Subcutaneously Embedded in Dairy Cows
Source: Animals (Basel). 2023 Nov 29;13(23):3700. doi: 10.3390/ani13233700 (PMC10705305; doi:10.3390/ani13233700)
Supplement: Supplementary file 1 [file animals-13-03700-s001.zip › animals-2653904-supplementary.pdf]

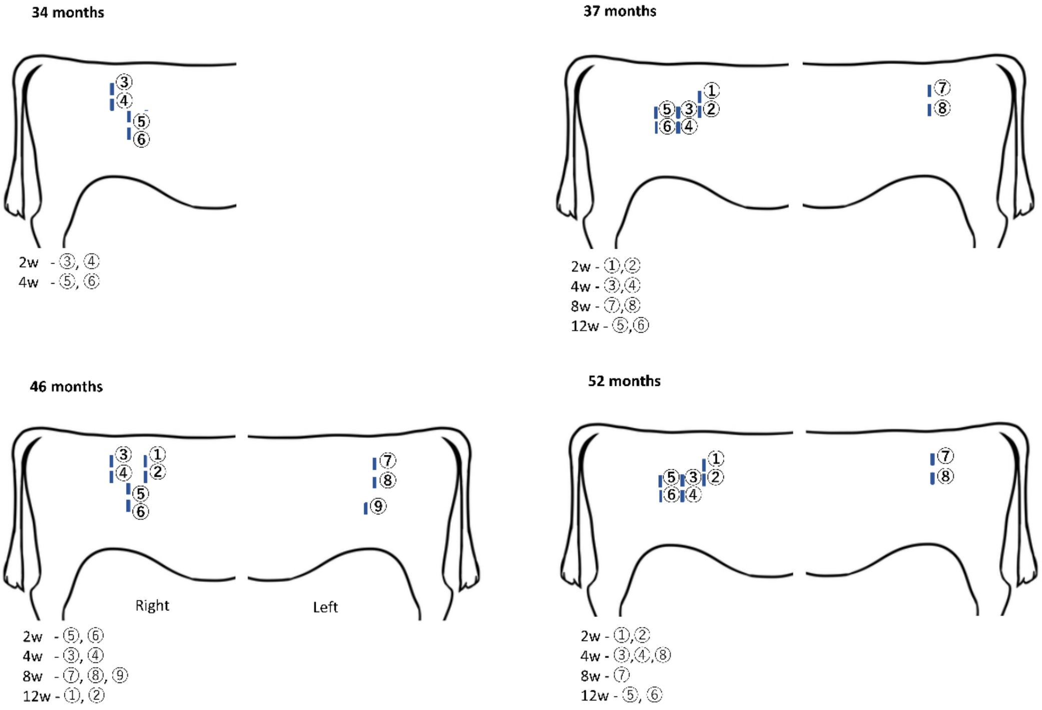


**Figure S1**. Cows and places of embedded implants used in this study. Four cows are used in this study. Embedded implants with connective tissues are removed and analyzed at weeks (w) 2, 4, 8, and 12.

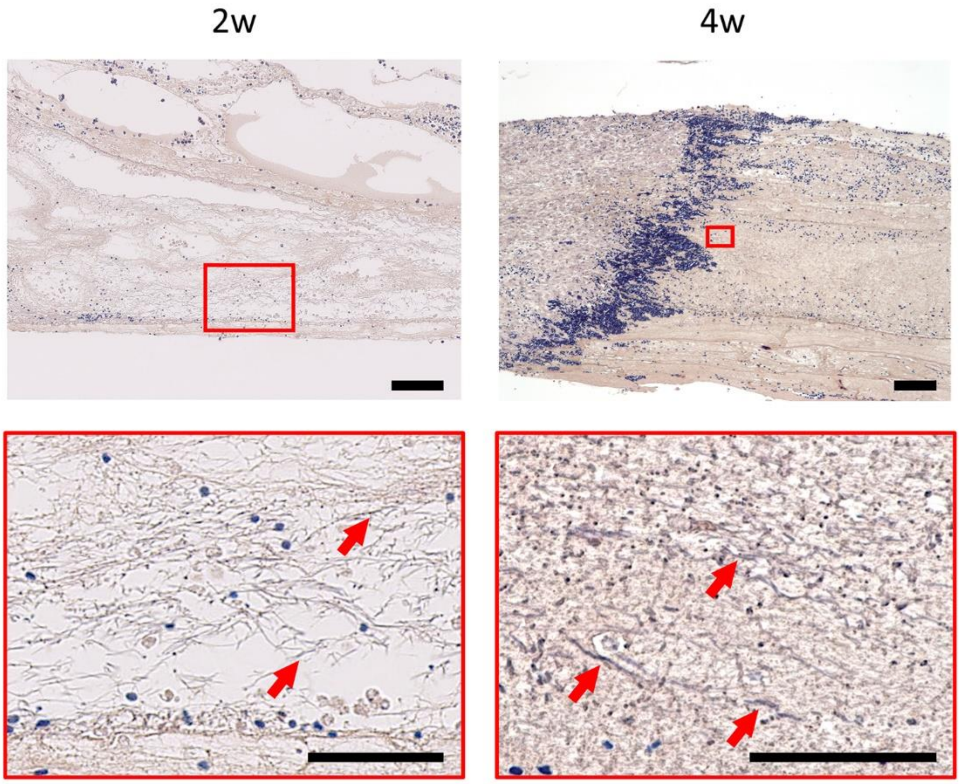


**Figure S2**. Fibrin fiber of connective tissue (CNT) formed around resin implants. Fibrin fibers comprising 2- and 4-week (w) CNTs are observed using phosphotungstic acid hematoxylin (PTAH) staining. Red squares indicating the edematous areas in the upper panels are magnified in the lower panels. Red arrows indicate observed fibrin fibers. Bars = 100 μm (upper panels), 50 μm (lower panels).

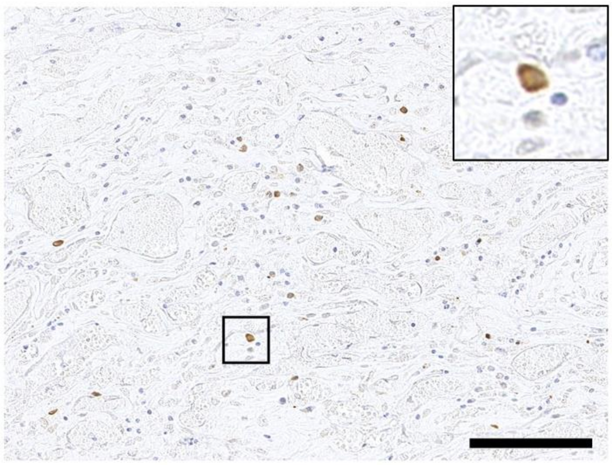

**Figure S3.** Immunohistochemistry of NOS2. NOS2+ M1-type macrophages are not observed in almost all samples but were observed in some connective tissues at 8 weeks. Bars = 100 μm
